# A rapid review of leading indicators to measure Australian farm safety culture

**DOI:** 10.1016/j.heliyon.2024.e32736

**Published:** 2024-06-11

**Authors:** Amity Latham, Jacqueline Cotton, Susan Brumby

**Affiliations:** a School of Medicine, Deakin University, 75 Pigdons Road, Waurn Ponds, Victoria, 3216, Australia; b National Centre for Farmer Health, Western District Health Service, Foster Street, Hamilton, Victoria, 3300, Australia

**Keywords:** Farm safety culture, Leading indicators, Measuring farm safety culture, Agriculture, Safety

## Abstract

Agriculture accounts for over half of Australia's land use therefore the farmers managing this land need to be safe at work. This paper offers measuring farm safety culture as a way to overcome the stagnation in the trend of fatal farm injury burden. To work towards achieving a way to measure farm safety culture, this research reviewed the leading indicators of safety. Following PRISMA guidelines, we screened for globally significant literature in the field of methodologies to measure safety climate and safety culture. We performed a rapid review of literature resulting in nineteen articles that add to our understanding of how to create and re-adapt existing questionnaires and produce validated metrics. The leading indicators were grouped into 8 dimensions where we found a translational disconnect between safety for organisational structures and safety for family farm businesses. This paper provides recommendations for government, safety regulators, policymakers, and industry of the leading indicators that may be applicable for measuring farm safety culture for Australian farmers.

## 1 Introduction

Agriculture accounts for over half of Australia's land use, therefore the management of land is important for both farm businesses and all Australians [1]. The farmers managing the land need to be safe at work, however the progress on reducing the fatal farm injury burden has stagnated over the past twenty years [2]. Australian agriculture has not been able to reduce fatality rates in line with other high-risk industries [3]. For example, in 2022, the fatality rate in the construction sector was 2.3 per 100,000 workers and for the agriculture, forestry and fishing sector the fatality rate was 10.4 per 100,000 workers, which is almost five times higher [3]. In Australia and globally, farm safety continues to be a priority area for safety regulators, policymakers, industry, and safety researchers, but it could be argued that the traditional ways to address farm safety is somewhat ineffective. The ways in which safety regulators and industry address farm safety hasn't changed much, and as a result, stakeholders are unanimously signalling for a new approach.

Meredith et al. [4] summarise the inequitable way in that agricultural incident data is presented and compared with other sectors, suggesting that general fatality rates are limited as they highlight the dangerous nature of farming occupations. Their article adds to the respective understanding of how farm fatality rates are calculated, particularly the complication of family farming and the at-risk populations – young people, non-paid workers, part-time farmers and bystanders. But even with a refined method of calculation, the safety sector remains reliant on lagging indicators to assess the safety behaviour of the agricultural workforce. Furthermore, agricultural work health and safety statistics are arguably meaningless to farmers, from whom safety stakeholder expect the change.

Lagging indicators are failure-focused measures [5]. They are a record of what has happened after an incident has happened. Individual accidents are said to occur in circumstances where the hazards are close to people and the defences are limited or non-existent [6]. Currently, industry remains reliant on lagging indicators such as injury and deaths [2,5], near misses, presentations to emergency departments, and days missed of work due to injury [7]. Added to this, knowledge of farm safety is imprecise. Data such as near misses goes unreported and presentations to emergency departments is not easily accessible [8].

Counter to lagging indicators are leading indicators. Leading indicators have been used by others as markers of evidence to collectively measure safety culture. They rely on human judgement of occupational health and safety (OHS) activity by pre-empting potential failure to enable identification, correction, and prevention of OHS incidents from occurring [5,9,10]. Leading indicators relating to farming are varied, and include conditions such as access to and use of personal protective equipment [11], exposure to farm-specific hazards [12,13] and working alone [14]. They can relate to objects, systems, attitudes and behaviours. Leading indicators switch the notion of measuring in hindsight to measuring in foresight.

The concept of safety culture is not new. Safety culture evolves gradually in response to local conditions, past events, the character of leadership and the mood of the workforce [6]. Safety culture could be considered as organisations’ social by-product, created by workplace attitudes that follow, evolve, and fasten to tasks. Mearns et al. [15] defines safety culture as what forms the environment within which individual safety attitudes develop and persist and safety behaviours are promoted. Safety culture is a leading indicator of OHS performance when it can be measured.

Fargnoli and Lombardi [16] describe safety climate as the combination of shared perceptions among workers on the procedures, practices, attitudes, and behaviours related to occupational safety. Farm safety climate is what is experienced by individuals and what influences and is influenced by cultures and management and operational systems. Griffin and Neal [17] define safety climate as an organisational factor and as an antecedent of systems safety.

Williamson et al. [18] enriched the field of safety climate and safety culture research underpinning the development of benchmarking instruments through measuring perceptions and attitudes about safety climate as an indicator of safety culture. Safety climate was defined as the safety ethic within an organisation or workplace that reflects employees’ or workers beliefs about safety and predicts behaviours in the workplace, while safety culture is referred more to the overall organisational and company-level beliefs and attitudes [11]. Benchmarking or measuring safety climate and safety culture within organisations with many employees became possible. Yet organisations with few employees, such as farms, are behind in these equivalent safety metrics.

Measuring safety culture or safety climate is complex as it requires quality data and consistent processes to achieve a meaningful measure. In this early stage of attempting to find methods applicable to farming, it was concluded that de-coupling the concepts of safety climate and safety culture would be too limiting. From herein, the term farm safety culture is a dual concept embracing farm safety climate.

The nearest attempt of measuring farm safety culture are farm safety checklists. Farm safety checklists are an accessible tool for Australian farmers to review the safety of their surroundings. In this review, we found 53 farm safety checklists. They were excluded from the articles of interest arguing the inconsistency in their form, lack of evidence of their use and validation, and inability to be recorded. At the same time, organisational safety culture is measurable on farms by using an appropriate combination of leading indicators to capture the culture [19].

Farming relies on the accurate collection and processing of data [20]. No different to crop yields, fleece weight and soil pH, measuring farm safety culture should be added to farmers’ repertoire to improve their performance and collectively measured across all farms. As described by Pollock et al. [11] in 2016 “a common concern among farmers is that they are implementing changes to their management systems, machinery and day-to-day farm management, but they have no feel for how well they are performing from a farm health and safety perspective … should the Authority conduct an inspection on their property”. This measurement should easily be adopted as data has already played an essential role in farming for more than a century [20]. This way, Australian agriculture can demonstrate and quantify the investment and impact of safety campaigns, health promotion activities, training programs, and other influential initiatives in farm safety.

The evolution in farm safety is to move away from lagging indicators as a measurement of occupational health and safety. Funded by Agriculture Victoria, through the Smarter, Safer Farms program, this work investigated how to measure farm safety culture. The rationale for this research is to better understand the leading indicators of safety culture that are fit-for-purpose for family farming businesses. This paper is structured to introduce the plethora of leading indicators that are used globally, if there is any relationship to Australian farming, and how as safety stakeholders we need to collaborate to measure safety culture, whilst identifying the existing gaps that may impede the measurement methodology for Australian farms.

## 2 Methodology

Given the infancy of measuring farm safety culture in agriculture, we needed a global perspective of the evidence in this field. This review followed the recommendations from the Cochrane Rapid Reviews Methods Group [21], particularly the rapid review method [22] to enable the development and synthesis of data and assess the work in the field. Initial search terms were farm*, agricult* labo*, risk*, safe*, approach*, innovat*, behavio*, evaluat*, and culture*. Each concept was searched independently and then combined using Boolean operators ‘AND’ and ‘OR’. Results from every search combination was recorded. The databases searched were MEDLINE Complete (via Ebsco), Embase (via Embase.com), APA PsycINFO (via Ebsco), Global Health (via Ebsco) and SocINDEX (via Ebsco). The search timeframe was limited to articles published from 2010 for manageability and relevance. Search comprehensiveness and literature sourcing was limited by language proficiencies (English). The PRISMA guidelines were followed for the database search process [23].

Once duplications were removed in EndnoteX9 the original bibliographic sources were exported to Covidence [24]. Two authors screened titles and abstracts and a third author resolved conflicts. The proportionate agreement was 94 % (0.93959). Articles were included if they demonstrated a methodology to measure safety, such as a survey or questionnaire, or a metric for safety culture. Articles were deemed ineligible when there was no full text, the subject was off-target, or the setting was unrelatable to agriculture or small business.

A grey literature search strategy was adapted to source government and other reports. We used Google Advanced search selecting the terms: assessment or tool or measure | “farm safety” | “safety climate” | “agriculture” | “program safety”| filetype:pdf. A total of 34,000 results were retrieved. The first one hundred websites, contained within the first 9 pages of search results met the project criteria; this was further refined for analysis (n = 75). An internal review of National Centre for Farmer Health's stakeholders' programs of interest also contributed to the preliminary dataset (n = 35). Upon screening, we found that over half of the grey literature was targeted at a farming audience (n = 57) with the majority being farm safety self-assessment tools (n = 53). These safety assessments are tools that end with the farmer and data cannot be collected from their use. As a result, these articles were excluded from those of interest.

The full search achieved a preliminary dataset of peer literature, grey literature and stakeholder programs of interest (n = 328). The authors included stakeholder materials and seminal papers written prior to 2010 for foundational theorising and interest [6,15,17,18]. The literature was tabled in discrete forms in Microsoft Excel with sub-headings to extract the data where applicable: web address, authors, date, organisation, source type, title, PDF download, target audience, study location, and evidence of measurement. Unique identifiers were based on the source (e.g. G = grey literature, P = peer reviewed material added by authors, S = stakeholder safety material and three or four-digit identification were retained from Covidence identifiers). The identifiers were used to file PDFs. Duplications were found across peer and grey literature (n = 6); both were retained and counted once.

**Fig. 1:**
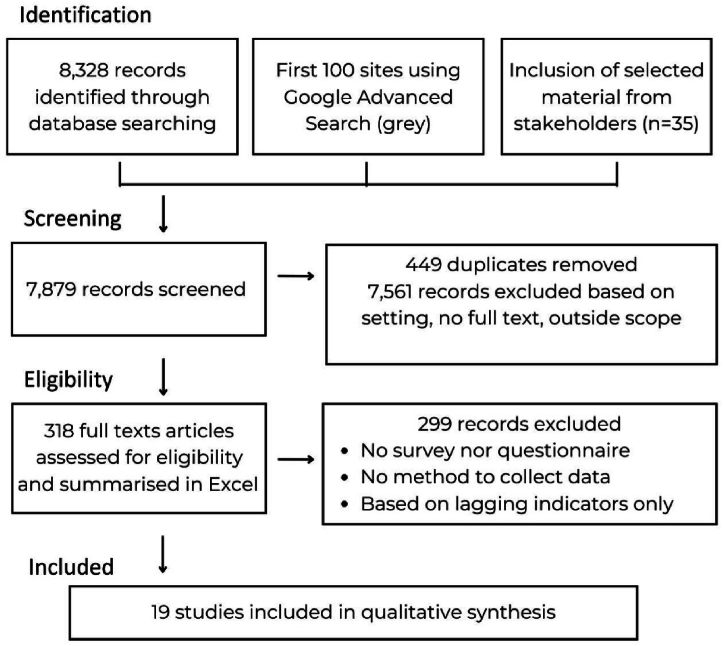
Publication selection for a systematic review [23].

In the final extraction phase, articles were refined to meet stringent criteria: (i) adds value to understanding the process of creating or re-adapting questionnaire; (ii) produced a validated metric; (iii) demonstrated potential and measurable leading indicators for agriculture. This resulted in 19 key articles of interest (Fig. 1).

## 3 RESULTS

The results from this rapid review demonstrate that there are numerous current safety culture questionnaires that use leading indicators. The literature, described and summarised in Table 1, shows a growing depth in understanding of leading indicators, that can be used in combination, to measure and benchmark workplace safety culture. There is inherent complexity within the structures of some of the questionnaires, which brings forth the questionable commitment required by the respondent to complete the survey and the commitment from the organisation to statistically analyse the results. The literature stems from a range of sectors, hence the authors have included commentary about each surveys’ relation or applicability to farm safety culture from an Australian perspective.

**Table 1:** Summary of key literature demonstrating leading indicators used to measure workplace safety culture.

ID	Author	Industry/Setting	Questionnaire structure	Relation for farm safety culture measurement	Country of origin
1P	Butterworth et al. [25]	Employed and unemployed	Household, Income and Labour Dynamics in Australia survey and Mental Health Inventory, covariate factors, 4 dimensions and 11 questions	Menial tasks on farm can also be related to weather or mechanical breakdowns that interrupt planned tasks.	Australia
4917	Chapman et al. [26]	Dairy	A survey instrument was re-adapted using a standardized mail survey, with 3 dimensions and 28 questions.	This survey is early evidence of re-developing a questionnaire specifically to measure safety culture on dairies.	USA
78G	Dairy Australia [7]	Dairy	Longitudinal survey seeking procedural evidence of worker health and safety.	Farm safety was integrated into the retention and capacity of the industry (paid and non-paid) and building on a longitudinal survey is ideal – such as this example	Australia
7660	Fargnoli and Lombardi [16]	Farming	NOSACQ-50 Safety climate assessment: 7 dimensions and 50 questions, with covariate factors. The result is mapped for visual interpretation.	This analysis adds to a database. It can be used in full or tailored. The questions are not designed for small organisations such as family farms as it is not ethically recommended for less than 20 respondents.	Italy
3137	Geng et al. [12]	Beef	WEST-AG is metric based on probability and consequence score. 15 dimensions of risk of injury were scored in Sweden and USA.	Limitations in the pilot suggest Australian beef farmers are most likely to have their own unique, or different, set of risk types as found between two comparison countries.	USA and Sweden
4P	Griffin and Neal [17]	Manufacturing and mining	A questionnaire consisting of 81 items. Two workplace surveys were undertaken, with the second survey revised and dimensions adjusted.	The data relied on archival records of large organisations for quality assurance auditing, which is not available in farming.	Australia
2331	Grimbuhler and Viel [27]	Vineyard	A literature review was used to scope the framework for a questionnaire resulting in 7 dimensions and 20 questions.	This approach is feasible for specifically measuring safety culture/climate for pesticide use in Australia, but it could be modified and applied more broadly.	France
5603	Irwin [14]	Farming	The questionnaire had 7 dimensions including covariate factors, the Big Five Personality Inventory, General Health Questionnaire, plus four dimensions.	The attitudinal elements of farm safety support researchers in recommending how safety training can be more engaging for particular personalities.	UK and Ireland
3765	Isaacs et al. [28]	Beef	Work Crew Performance Model (WCPM) includes 32 critical action factors in 4 categories.	The process created a cattle safety handling checklist used with success in Master Cattleman educational workshops for Kentucky farmers. Safety checklists in cattle handling could be advocated by Meat and Livestock Australia (MLA).	USA
2P	Lay et al. [29]	Employed persons (>15 h per week)	Covariate factors and ethnicity with job tenure, together with the Hazard and Vulnerability Questions containing 4 dimensions and 27 questions were used to improve measurement and evaluation of how workplace context impacts individual risk of injury or illness.	Knowing how labour market sub-groups experience vulnerability can inform better-tailored primary prevention interventions. There is potential to adapt this survey to CALD workers in intensive animal production and horticulture sectors in Australia.	Canada
2325	Leppälä et al. [13]	Equine	The InnoHorse accounted for externalities and internal risks that influence 7 dimensions of safety with 41 checks for stable managers.	The process provides a practical model of a needs-driven, and statistically validated, project to address a safety issues around large animals but lacks a mechanism for data collection.	Finland and Sweden
22S	Lingard [30]	Construction	A multilevel questionnaire for workers has 9 dimensions across 3 levels (organisation or principal contractor, the project, and the workgroup) to measure climate at any given time.	The tool is designed to measure multiple groups which can be assimilated with farmers relying on contracting services (e.g. baling, harvesting, windrowing etc.) where outsiders may bring a different safety culture to the organisation.	Australia
16G	Pollock et al. [11]	Farming	The benchmarking had 3 dimensions: 20 safety climate questions, 35 safety management questions, 15 control of major hazard questions on farms. The research uses total factor productivity (TFP) to test the scores in each sector to look for trends of lost productivity.	This is a superior and validated Australian example of how to measure safety climate, safety management systems and control major safety hazards on farms as a triangulation of comparable scores, and to use the scores to prioritise interventions.	Australia
23S	Safe Work Manitoba [31]	Employed persons	The tool comprised of 12 questions to self-assess workplace's safety culture using a Likert scale and colour coded scale of results.	The short tool with instant feedback has merit for farmers to track their organisational position. Covariate factors and questionnaire results need to be ethically captured from participants for research.	Canada
3P	Shea et al. [32]	Multiple industries (n = 6)	The OPM- MU survey is constructed of leading indicators of OHS performance encompassing 10 questions.	The survey could be an initial ‘flag’ of the leading indicators of OHS as it has the potential to be a benchmarking tool for workplaces both within and across organisations. The questions may need to reflect farming for industry engagement.	Australia
39S	Schirmer [33]	Farming	There are 7 dimensions of wellbeing. One specific dimension was developed to address workplace safety on farms and individual and community safety culture.	This survey is most applicable to agriculture particularly those designed to collect data to measure health, safety and wellbeing on farms.	Australia
5G	Leppälä [34]	Farming	To measure safety behaviour the survey included covariate factors, 3 questions in injury history, 17 questions on safety practices, 35 questions on attitudes, 5 questions on community safety culture, and 7 questions on obstacles to being safe.	This Sacurima COST survey is most applicable, noting that all of the participating EU countries have adjusted it to their own language, and operating environment.	33 European countries, plus international participants
5160	Terjék [35]	Farming	Three questionnaires were developed: General questionnaire – 72 questions; Farm leader questionnaire – 22 dimensions with 178 questions; and the Farm worker questionnaire – 26 dimensions with 171 questions.	The analysis of influential material factors and infrastructural challenges, such as tractor age, shows applicability for Australian farming.	Hungary
73G	Whitman and Clark [36]	Dairy	The Vital Capital Index is structured as 3 dimensions: Prosperity, People and Plant. Farmers are given guidelines of how to score each criterion for self-assessment of their culture.	This is an applicable and in-depth benchmarking tool for dairy farmers that demonstrates a way of self-assessing farm culture, not only OH&S.	USA

Contemporary work health and safety research is recognising these leading indicators as a mechanism to prevent workplace injury and fatality. It should be noted that few safety culture researchers have achieved a final metric. The belief, and hence the process, of farm safety culture measurement is in its infancy. In most cases the evaluation of safety programs and initiatives is relatively short-term and evaluates the process, more so than the outcome.

The term – dimension – is given to a theme and/or group of similar leading indicators. These dimensions are broad, but looking closer at each singular leading indicator their differentiations are more nuanced. These dimensions share associations with preventing fatalities, injury and illness. Whether they are agriculturally related or generic in nature, they are the signs and signals of a safe workplace culture. There were 8 dimensions identified and these are discussed below. These dimensions measure safety culture at individual and organisational levels globally.

### 3.1 Dimension 1. Management and administration

The role or activity of management is a common and multi-faceted leading indicator. Communication and feedback are generally provided by managers. The implementation of workplace rules and enactment of safety processes through accountability, commitment and leadership are relational to farm safety culture. Shea and colleagues [32] identified features of leading indicators of occupational health and safety and then examined the literature on the measurement of leading indicators, selecting and adapting the Organisational Performance Metric (OPM) [37]. The OPM was developed at the Institute for Work & Health in Canada and designed specifically to measure leading indicators of OHS. Shea and colleagues [32] adapted and psychometrically evaluated the tool in Australia, creating the Organisational Performance Metric – Monash University (OPM-MU). This short tool has the potential to benchmark workplaces encompassing a range of management-level leading indicators. The H&S Climate Assessment Tool surveys the principal contractor, the project, and the workgroup in the construction sector [30]. A ‘pool’ of potential questions is relatable to management and administration, within dimensions of leadership, communication, organisational goals and values, supportive environment, responsibility and learning. The framework by Griffin and Neal [17] included safety communication, safety practices and manager's values as leading indicators of organisational safety climate. Lay and colleagues [29] include a dimension specifically for policies and procedures to measure training, communications, systems to deal with hazards, presence of a safety committee or representative, and investigations.

The Power of People on Australian Dairy Farms [7] is a longitudinal three year study of the dairy workforce. This survey measures the use of, and updates to farmers' written health and safety plans, as well as the implementation of written or informal operating standards for tractors. Farmers are profiled to recognise a micro-workforce on their farms. In their pesticide risk awareness work Grimbuhler and Viel [27] ask respondents six indicators concerning management's commitment to pesticide safety and three indicators of knowledge and processes following pesticide exposure incidents.

The Australian Centre for Agricultural Health and Safety established a longitudinal study of 335 NSW farm enterprises to derive data on farm health and safety management. The specific objective was to develop scores for measures of Safety Climate, Safety Management Systems and Control of Major Hazards, and to try to explain the determinants of those scores [11]. The questions reflecting the dimensions of managing farm safety included the engagement of workers and management in safety on the farm, assessment of hazards and risks, safety plans and actions, information, training and resources on workplace safety and systems, and monitoring and recording of health and safety incidents, situations and processes [38].

### 3.2 Dimension 2. Prevention of injury strategies

Preparedness and prevention activities are leading indicators. Chapman and colleagues [26] included self-protective activities in their questionnaire. This dimension included annual inspections for hazards, structures and storage, the use of an inspection form, and scheduling of safety meetings with staff and family members. The Power of People on Australian Dairy Farms [7] also includes the involvement of staff in safety scans and hazard identification.

The InnoHorse web tool was developed to support horse stable managers in business, safety, pasture and manure management [13]. Following a review of lagging indicators (i.e., insurance claims, horse-related injuries) the Horse Stable Safety Map framework focussed on incident prevention activities measured through 7 dimensions and 41 checks for stable managers. The areas to check safety included walkways and corridors, safety of building facilities, ergonomics, tools and machines, PPE, fire safety and rescue planning, and employee and customer safety, plus four miscellaneous leading indicators [13]. This paper was included for its process; evidence suggests like most farm checklists it cannot collect data.

In the Australian Centre for AgHealth and Safety study of NSW farm enterprises, the Control of Major Hazard questions (n = 15) related to actual processes and practices on the farm enterprise [11]. These hazards were selected by Farm Safe Australia as key priorities areas based on incident data: tractors, PTOs, augers, residual current devices (RCDs), chemicals, silos, safe play areas for children, vehicle safety, helmets and PPE [11].

### 3.3 Dimension 3. Perceptions of safety

Fargnoli and Lombardi [16] applied the NOSACQ-50 questionnaire in an agricultural context to measure shared perceptions of management and workgroup safety related policies, procedures and practices [39]. The NOSACQ-50 measures safety climate; the leading indicators are perceived conditions that contribute to individuals' motivation as well as relation to occupational safety [39]. The dimensions include measures of management's priorities, competence, commitment, safety empowerment and safety justice and worker's safety commitment, priority and risk non-acceptance, communication and trust [16]. For Irwin and Poots [14] safety motivation assessed the extent to which farmers felt safety was an important part of their work, and safety compliance indicated the extent to which participants complied with safety regulations. To analyse how resources and procedures shape risk for certain labour markets Lay and colleagues [29] measures employee's awareness and employee's empowerment in the workplace.

In the study by Pollock, Fragar and Griffith [11] longitudinal data was derived on farm health and safety management and how it relates to farmer perceptions. Slightly reworded, but based on the work of Williamson [18], five dimensions measured personal motivation for safety, positive safety practices, risk justification, fatalism and optimism. The questionnaire by Butterworth and colleagues [25] sought job-related leading indicators in four dimensions including job demand and complexity, job control, job security and effort, reward and fairness. This Psychosocial Job Quality survey measured workers’ mental wellness to occupational health and safety.

The Safety Culture Assessment is a tool which enables data to be collected as part of the Safe Work Manitoba certification program [31]. Feedback is instant for this generic workplace safety culture measurement. There are 12 questions seeking a delegate to rank their perceptions of the organisation's routine safety audits, safety values, provision of safety information, and management's involvement in the safety program, recognition of safe acts, prevention, communication and trust.

### 3.4 Dimension 4. Exposure to hazards

Inverse to prevention, Geng and colleagues [12] and Lay and colleagues [29] use exposure as indicators of safety culture. The WEST-AG is a metric based on probability and consequence [12]. Following field observations, the second phase is a linear-scale assessment of exposure to risk of injury. The questions include, but are not limited, to exposure to machinery, struck by flying objects, overexertion, using hand tools, poor housekeeping, trips from uneven terrain, chemical exposure, burns, frostbite, and contact with dangerous animals [12]. These farm-based leading indicators provide early warning signs of potential incident. As exposure is reduced, they may promise an improved gauge of work health and safety activity. Lay and colleagues [29] measure exposure to hazards over time with specific questions relating to generic occupational health such as “how often do you have to work in a bent, twisted or awkward position?”

The Work Crew Performance Model rests on a set of assumptions about the nature of the worker and the job [28]. Thirty-two critical action factors were identified and categorised as four dimensions: environmental conditions, animal behaviour, handling equipment and facilities, and handling techniques [28]. The Regional Wellbeing survey asked survey participants if, in the last 12 months, they had experienced any of a number of known WHS risks, including long working hours, work stress, bullying/harassment, work related injury or illness, engaging in unfamiliar tasks or using equipment that was poorly maintained, and near misses [33]. The use of PPE and engineering controls as protection strategies are measured in the Power of People on Australian Dairy Farms [7] which measures changes in ROPS on quadbikes and helmet wearing over time. The questionnaire by Griffin and Neal [17] included safety equipment.

### 3.5 Dimension 5. Personality, attitude and behaviour

Questionnaires may target individuals’ sense of self, sense of others, and sense for the safety culture of the organisation. Griffin and Neal [17] included compliance motivation, participation motivation, safety compliance, and safety participation as attributes to measure safety climate. For farmers handling pesticides Grimbuhler and Viel [27] included dimensions grouping safety compliance, safety participation, and teamwork climate as leading indicators. Irwin and Poots [14] assessed non-technical skills by working as a team compared with working alone. They measured situation awareness, teamwork and communication, leadership, task management, and decision making [14].

The H&S Climate Assessment Tool consists of 9 dimensions; 7 of which are led more so by management or through administration [30]. The dimensions relating more to personality and attitude were trust in people and systems, and resilience. To measure safety behaviour the Sacurima COST survey asks for respondents’ obstacles to safety behaviour, including tiredness, stress, workload, and weather conditions. Questions were grouped under injury history, safety practices, attitudes and intentions, and safety culture in the community [40].

### 3.6 Dimension 6. Safety and financial considerations

The willingness to pay or the ability to afford improvements in safety is a leading indicator, particularly for small to medium business. Irwin and Poots [14] attribute workload, costs and profit margins, problems caused by weather or equipment, and work-life balance as stressors in one dimension. Chapman and colleagues [26] ask respondents “how much would you be willing to pay for an inspection by an outsider?”, and “how much would you annually budget for hazard corrections?” The Vital Capital Index scored dairy producers’ quality of life as an essential component if dairy farms are to be sustainable. Quality of life includes economic standard of living, as well as job satisfaction, personal health, time for family, friends, and leisure as well as achieving life goals [36].

### 3.7 Dimension 7. Safety training

Designed for mining and adapted by Isaacs et al. [28] as a safety framework for cattle handling, the Work Crew Performance Model (WCPM) seeks to define performance variability within similar tasks [28]. As a result, it offers an improved method of measuring training effectiveness and evaluating workers’ performance within the practical confines of the systems [28]. The questionnaire by Griffin and Neal [17] and the OPM-MU [32] both included safety training as a leading indicator.

### 3.8 Dimension 8. Knowledge of workplace rules

Chapman and colleagues [26] assess knowledge of rules and regulations. Their questionnaire checks that extra riders are not allowed in tractors and that seatbelts are worn while operating vehicles on public highways. The questionnaire by Griffin and Neal [17] included safety knowledge as an attribute in their framework linking safety climate to safety performance. Grimbuhler and Viel [27] include rules and best practice and knowledge as dimensions. The farmers handling pesticides were asked if they knew safety regulations and if they knew when to use PPE [27]. In their non-technical skills approach, Irwin and Poots [14] attribute risk tolerance to assess the extent to which farmers were prepared to bend the rules, or take shortcuts to achieve performance targets.

## 4 Discussion

Measuring safety culture on farms is complex as many farm businesses are family owned and they may consist of one owner/operator with unpaid family support. These small family-run organisations means that measuring safety culture is at a micro-scale bringing with it significant risk of skewing the metrics and the challenge of being a “hard to reach” population [41]. This small survey sample makes the questionnaires by Griffin and Neal [17], and Lay and colleagues [29] not fit for use within the farming context.

The heterogeneity among farm businesses and safety climates means there is also diversity in safety culture. Despite the previous argument to fuse safety climate and culture as collective, at any point in time every farming business will have its own safety climate that will measure differently to others. The uniqueness of farming brings with it significant challenges therefore only the leading indicators that measure farm safety culture, rather than climate, should be selected. This requires identifying who to work with, where to begin, and which indicators to measure.

There is currently little collective action from farming groups, health services, specific farming districts, government organisations or agriculture sectors to longitudinally quantify farm safety attitudes and behaviours. There remains a knowledge gap regarding the long-term effects of safety-specific interventions and on-farm tools and their impact on farm safety culture. At the same time, there is also significant and available data from many sectors plus the statistical capacity to connect multiple sets. As demonstrated in the literature, databases have the potential to be the denominator in the metric to measure farm safety culture, however there is no fit-for-purpose database to achieve this completely.

Databases can overcome the persistent surveying of individuals to capture a meaningful sample. Farmers are often inundated with surveys therefore using existing databases not only collects more information, but it reduces survey fatigue and resourcing. Evidence suggests that measuring farm safety culture is generally stronger in theory than practice. It needs to be noted that few safety culture researchers have achieved the final metric. There have been a number of attempts to develop methodology that can accurately and longitudinally measure safety culture yet very few have proven able to present a factor structure that is consistent in different contexts, and many have a vague theoretical grounding [39]. At this early point in measuring safety culture within Australian agriculture, much can be learned from other sectors as to how to link databases and build efficiencies to reduce costs. The use of a validated questionnaires reduces the need for re-invention. Experts, stakeholders, and peak industry may be used for critical problem solving and providing different perspectives and play a part in the broader validation process to increase the likelihood of the questionnaires’ success. A recommendation is to seek new linkages and to build on a validated questionnaire to create longitudinal baseline information.

Data that focusses on leading indicators is not effectively collected demonstrating the disconnection between farm safety organisations and farming populations. This was shown when the authors excluded over half the grey literature early in the review as farm safety checklists do not collect nor share data. Whilst success has been seen in other high risk industries measuring safety culture, this review highlights the need to translate the notion of farm safety culture from theory to practice. The gap in current knowledge confirms that it is unknown whether the methods for measuring farm safety actually makes a difference to people's lives living and working on Australian farms.

To achieve a way to measure farm safety culture the enactment of this data collation requires significant engagement from government to introduce this concept across the industry more broadly. It is an ongoing process that will require funding to re-measure the influence of interventions at farm-level. A collaborative and inter-sectoral approach for safety research, farm extension, and data management Australia-wide, will maintain a strong farm safety presence into the future.

### 4.1 Limitations

The discussion section demonstrates the broader limitations of measuring farm safety culture. This research is limited to a desktop-study that relies on the pre-conceptions of leading indicators used in other contexts as foresight into OHS performance. There is no evidence of these indicators being challenged in Australian family-owned farms settings, therefore current leading indicators may not be fit-for-purpose nor relevant. Insurance claims for farm machinery repairs, animal welfare, mental health, farmers’ exposure to pesticides, days spent off farm for professional development, and age of operational tractors may be some examples of more farming-sensitive and production-based leading indicators, which would need to be used as a matrix, to measure farm safety culture. This study did not set out to test leading indicators applicability in the field.

## 5 Conclusion

The progress on reducing the fatal farm injury burden has stagnated in Australia and agriculture has not been able to reduce fatality rates in line with other high-risk industries [3]. It could be argued that the epidemiology of deaths and injuries on farms is difficult for farmers to relate to which is why safety regulators, policymakers, industry and safety researchers are unanimously signalling for a new approach. As a result, this paper has reviewed literature in the field to offer the solution through the on-going measurement of farm safety culture at farm-level.

The Australian agricultural sector will advance when the combination of leading indicators that effectively measure farm safety culture, over time, are determined. It is recommended that the farm safety culture metrics must inform, encourage action, be replicable across sectors, reportable, and transparent in collection. This also requires maintaining strong stakeholder, industry and government support. The methodology to measure farm safety culture requires ownership and must be embraced and understood by those working closely with farmers. Development and incorporation of safety culture metrics into the on-going evaluation of farm safety extension and intervention will facilitate the longer-term measurement of their impact on both lag and leading indicators of safety. It is important for safety stakeholders to progress the way we address farm injury and fatality. We need to overcome the stagnated trend of farm injuries and fatalities as witnessed in the past twenty years in Australia, and globally. Measuring farm safety culture demonstrates a new attempt, and new possibilities, of making farms safer places.

## Data availabilty statement

Data for this review is unavailable.

## CRediT authorship contribution statement

**Amity Latham:** Writing – original draft, Data curation, Conceptualization. **Jacqueline Cotton:** Writing – review & editing, Validation, Methodology, Funding acquisition, Conceptualization. **Susan Brumby:** Writing – review & editing, Supervision, Project administration, Funding acquisition, Conceptualization.

## Declaration of competing interest

The authors declare that they have no known competing financial interests or personal relationships that could have appeared to influence the work reported in this paper.
